# Genetic diversity and structure in the Endangered Allen Cays Rock Iguana, *Cyclura cychlura inornata*

**DOI:** 10.7717/peerj.1793

**Published:** 2016-03-10

**Authors:** Andrea C. Aplasca, John B. Iverson, Mark E. Welch, Giuliano Colosimo, Evon R. Hekkala

**Affiliations:** 1 Department of Biological Sciences, Fordham University, New York, NY, United States; 2 Department of Biology, Earlham College, Richmond, IN, United States; 3 Department of Biological Sciences, Mississippi State University, Mississippi, MS, United States; 4 Current affiliation: College of Veterinary Medicine, Cornell University, Ithaca, NY, United States

**Keywords:** Allen cays rock iguana, Island conservation, Reintroduction, Genetic structure

## Abstract

The Endangered Allen Cays Rock Iguana (*Cyclura cychlura inornata*) is endemic to the Allen Cays, a tiny cluster of islands in the Bahamas. Naturally occurring populations exist on only two cays (<4 ha each). However, populations of unknown origin were recently discovered on four additional cays. To investigate patterns of genetic variation among these populations, we analyzed nuclear and mitochondrial markers for 268 individuals. Analysis of three mitochondrial gene regions (2,328 bp) and data for eight nuclear microsatellite loci indicated low genetic diversity overall. Estimates of effective population sizes based on multilocus genotypes were also extremely low. Despite low diversity, significant population structuring and variation in genetic diversity measures were detected among cays. Genetic data confirm the source population for an experimentally translocated population while raising concerns regarding other, unauthorized, translocations. Reduced heterozygosity is consistent with a documented historical population decline due to overharvest. This study provides the first range-wide genetic analysis of this subspecies. We suggest strategies to maximize genetic diversity during ongoing recovery including additional translocations to establish assurance populations and additional protective measures for the two remaining natural populations.

## Introduction

Invasive species, anthropogenically mediated sea level rise and stochastic natural events such as hurricanes disproportionately affect island populations (Spiller, Losos & Schoener, 1998; Schoener, Spiller & Losos, 2004). Insular species are less likely to recover from such events due to the demographic and genetic attributes of naturally small populations. In particular, reduced effective population size and genetic diversity may constrain the ability of small, isolated populations to recover after such impacts.

Iguanas of the genus *Cyclura* inhabit two main island groups in the West Indies: the Greater Antilles and the Bahaman archipelago. Although taxonomic classifications remain controversial, of the 10 species and 15 subspecies identified (ITWG, 2011), most are classified as Endangered or Critically Endangered by the International Union for the Conservation of Nature (Alberts, 2000; Knapp & Pagni, 2011). *Cyclura* spp. are these islands’ largest native extant vertebrates and perform a key ecosystem role as herbivores and seed dispersers (Iverson, 1985). However, loss of habitat, introduced mammals, harvesting by humans for food, and poaching for the pet trade have severely impacted populations (Alberts, 2000). Additional threats are posed by climate-related sea level rise and an increase in stochastic weather events. These factors can radically reduce population numbers in a short period of time, potentially leading to an “Extinction Vortex,” wherein extrinsic factors contribute to small population size, at which point intrinsic factors such as inbreeding further reduce individual fitness (Caughley, 1994; Frankham, 1998; Brook, Sodhi & Bradshaw, 2008). Hence, measures of genetic diversity and inbreeding can reveal whether a population may be particularly vulnerable to local extirpation (Frankham, 2005).

Within the Bahamas archipelago, the Allen Cays Rock Iguana (*Cyclura cychlura inornata*), currently classified as Endangered by the IUCN (Alberts, 2000; Blair & West Indian Iguana Specialist Group, 2000), is one of three subspecies of *Cyclura cychlura* that also includes the Andros Island Rock Iguana (*C. c. cychlura*) and the Exuma Island Rock Iguana (*C. c. figginsi*). *Cyclura c. inornata* was believed extinct due to human harvest when it was originally described (Barbour & Noble, 1916). However, the subspecies was rediscovered, and is known from the Allen Cays, a few small islands in the northern Exuma Island chain of the Central Bahamas (Schwartz & Carey, 1977). For the past 35 years, *C. c. inornata* has been actively studied and population density is quite high on some islands (Iverson et al., 2006). It has been the subject of active management, including translocation, in an effort to establish an assurance colony in the nearby protected Exuma Cays Land and Sea Park (Knapp, 2001). Recently, human activities have again impacted populations of Allen Cays Rock Iguana (Smith & Iverson, 2006; Smith & Iverson, in press; Knapp et al., 2013); however, the magnitude of the effects on iguanas of natural disturbances, such as hurricanes, and periodic drought has not been studied.

Despite these current threats, the two natural populations of *C. c. inornata* (Leaf Cay and U Cay) have increased steadily over the last fifty years, with current combined populations of approximately 1000 individuals, with each population likely approaching carrying capacity (Table 1) (Iverson et al., 2006). Poaching for human consumption and the illegal pet trade still occurs, and human access to the Allen Cays has resulted in the introduction of non-native house mice (*Mus musculus*). Strong indicators suggest that the presence of rodents negatively affects iguana populations (Hayes et al., 2012). Because of this concern, the mice on this Cay were recently eradicated (Jones, 2010; Bahamas National Trust, 2013).

**Table 1:** Primer pairs used to sequence three separate mitochondrial DNA regions (ND4-LEU, cytochrome b (cytB), and Control Region (CR)) for Allen Cays Rock Iguana.

Primer name	Sequence	Reference
ND4	CAC CTA TGA CTA CCA AAA GCT CAT GTA GAA GC	Arévalo, Davis & Sites (1994)
LEU	CAT TAC TTT TAC TTG GAT TTG CAC CA	Arévalo, Davis & Sites (1994)
L14724 (cytb)	CGA AGC TTG ATA TGA AAA ACC ATC GTT G	Irwin, Kocher & Wilson (1991)
H15149 (cytb)	AAA CTG CAG CCC CTC AGA ATG ATA TTT GTC CTC A	Kocher et al., (1989)
trnP21F (CR)	CCC CCA TCT CCA GCC CCC AA	This study
trnP29F (CR)	TCC AGC CCC CAA AAC TGG CA	This study
DL463F (CR)	CGA CTA AGT TAT CTG GCA AAA CGC GA	This study
DL742R (CR)	TGT GGG ACT GAA GCC AAC CCC	This study
trnF45R (CR)	GCG GCA TTT TCA GTG CCG TGC	This study
12S70R (CR)	CAC TGG TGT GCG GAT GCT TGC	This study

The introduction of non-native species remains a threat to small insular lizard populations. For example, the introduction of feral dogs and cats nearly extirpated a population of 5,000 Turks and Caicos Iguanas (*Cyclura carinata*) in less than three years (Iverson, 1978), and a single introduced Raccoon (*Procyon* sp.) was linked to a 35–67% decrease in the only existing population of White Cay Iguana (*Cyclura rileyi cristata*) in the Bahamas within a year (Alberts et al., 2002; Hayes et al., 2004). Gasc et al. (2010) linked a decrease in genetic diversity in the insular lizard *Anolis sagrei* to an invasion of *Rattus* sp. In addition, daily human contact in the form of tourism and supplemental feeding has been linked to a breakdown of natural iguana behavior (habitat use, and food preferences), and altered blood chemistry, in the Allen Cays Iguana (Iverson et al., 2006; Hines, 2011; Knapp et al., 2013).

Here, we examine patterns of genetic diversity and structuring among these small, insular populations of the Allen Cays Iguana (*C. c. inornata*) to better inform conservation management planning for the northern Exumas. Specifically, we (1) assess genetic relatedness among populations; (2) identify the likely origins of recently discovered translocated populations; and (3) evaluate the potential for natural dispersal, and translocation to aid in population persistence and resilience in the face of increasing anthropogenic impacts. The current study investigated twelve DNA microsatellite loci and three mitochondrial DNA regions to examine genetic diversity in *C. c. inornata* across the known (natural and translocated) distribution of the subspecies in the Exuma Islands (Iverson & Mamula, 1989; Iverson, Hines & Valiulis, 2004; Smith & Iverson, 2006; Iverson et al., 2006).

## Methods

### Sample collection and estimation of population size

**Figure 1:**
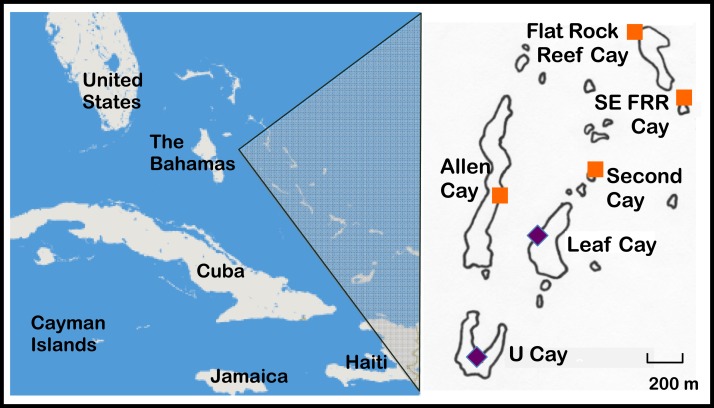
Naturally occurring populations of the Allen Cays Rock Iguana (*Cyclura cychlura inornata*) are found on two cays in the Northern Bahamas, Leaf and U Cay, marked by the symbol, 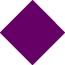. Islands with recently documented iguanas (Allen, Flat Rock Reef, Southeast Flat Rock Reef, and Second Cay) are marked by the symbol, 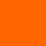. The experimentally translocated population on Alligator Cay located farther south in the Exuma Island Chain of the Bahamas is not shown. (Adapted from Hines, 2011; Iverson, Hines & Valiulis, 2004.)

The Allen Cays are a small group of islands located in the northern Exuma island chain in the Bahamas (Fig. 1, Schwartz & Carey, 1977). Most iguanas (>85%) in the Allen Cays are now uniquely identifiable by a combination of toe clips and passive integrated transponder (PIT) tags due to an ongoing field study that was initiated in 1980. Samples were collected and processed under permits from the Bahamas National Trust, the Bahamas Environment Science and Technology (BEST) Commission, the Exuma Cays Land and Sea Park, and the Bahamas Department of Agriculture.

We collected tissue samples from 285 unique individual iguanas during fieldwork conducted between 2006 and 2012 from seven cays (each <7 ha) in the Exumas that collectively comprise the entire known range of the Allen Cays Rock Iguana (Table 1). Tissue samples collected were in the form of blood or toe clips. Blood samples were drawn from the caudal vein of previously marked iguanas, stored in a blood lysis buffer (0.1 M Tris–HCl, 0.01 M NaCl, 0.1 M EDTA, 1% SDS) at a ratio of 1:2 (blood to buffer) at ambient temperatures during the field-work and at −20 °C upon arrival to the laboratory. We marked unidentified iguanas with a unique combination of toe clips, and toe clip samples were stored in 85% ethanol at ambient temperatures. Approximately 50% of subadults and adults from Leaf and U Cays are captured each year, and recapture rates of marked animals vary between 85% and 90%. Hence our population size estimates are quite reliable (see also Iverson et al., 2006).

### Laboratory methods

We extracted genomic DNA from blood and tissue using Qiagen DNeasy Blood and Tissue Kits (Qiagen, Inc., Valencia, CA, USA) and an Applied Biosystems 6,100 Nucleic Acid PrepStation (Applied Biosystems, Carlsbad, CA, USA) following manufacturer’s instructions. Final elutions of DNA were stored at −20 °C.

We sequenced 95 of the 285 individuals sampled for three mitochondrial loci (NADH-4, CytB, and Control Region). These 95 individuals included representatives from all sampling sites (Table 1) to assess mtDNA variation among islands. We sequenced a portion of the NADH-4 (903 bp), and CytB (425 bp) loci using published primers (Arévalo, Davis & Sites, 1994; Irwin, Kocher & Wilson, 1991). We designed additional primers to target 1,000 bp of the control region (Table 2). We amplified target regions using Illustra Ready-To-Go PCR beads (GE Healthcare, Pittsburgh, PA, USA). Total reaction volumes were 25.5 µL including ∼15 ng of template DNA with forward and reverse primer concentrations of 10 pmol each. Thermal cycler conditions included a 2 min initial denaturation at 96 °C, 35 cycles of 96 °C for 30 s, 50 °C for 30 s and 72 °C for 1 min, and a final extension at 72 °C for 10 min.

**Table 2:** Estimated census population sizes (*N*), island sizes (*ha*), expected heterozygosity (*H*_*E*_), inbreeding coefficients (*F*_*IS*_), Allelic richness (*α*), number of private alleles (*A*^*P*^), and effective population sizes (*Ne*) based on eight polymorphic microsatellite loci for known Cays supporting the Allen Cays Rock Iguana (*Cyclura cychlura inornata)* in the Exuma Islands, the Bahamas. Leaf and U Cays (*) are presumed natural populations.

Cay	Est. Pop.	Cay Size (*ha*)	*mtDNA* n	*nDNA* n	*H*_*E*_	*F*_*IS*_	*α*	*A*^*P*^	*Ne*
Leaf*	700	4	20	107	0.37	−0.02 (*p* = 0.71)	1.83	1	27.8
U*	300	3	20	77	0.42	−0.07 (*p* = 0.95)	1.98	1	21.9
Alligator	75	1.8	9	9	0.35	−0.23 (*p* = 0.93)	1.76	0	30
FRR	200	5.3	20	46	0.46	−0.12 (*p* = 0.98)	2.09	0	∞
Allen	22	6	19	22	0.38	−0.05 (*p* = 0.74)	1.85	0	10.1
Second	3	0.5	3	3	0.42	−0.56 (*p* = 1.00)	1.88	0	∞
SEFRR	<8	0.5	4	4	0.13	0.31 (*p* = 0.42)	1.31	0	2.2

PCR products were cleaned using AMPure magnetic beads (Agencourt, Beverly, MA, USA), and cycle-sequenced using BigDye Terminator v 3.1 Kit (Applied Biosystems, Carlsbad, CA, USA). Sequencing products were purified using CleanSeq (Agencourt, Beverly, MA, USA) and run on an ABI 3730xl DNA Analyzer (Applied Biosystems, Carlsbad, CA, USA). We aligned and edited sequences using Sequencher 5.0 (Gene Codes Corporation, Ann Arbor, MI, USA).

We attempted to genotype all 285 individuals at 12 microsatellite loci (Table 3) previously developed for other taxa in the genus *Cyclura* (Malone et al., 2003; Lau et al., 2009; Welch et al., 2011). These 12 loci were scored using standard PCR based methods, and were selected because they were found to be polymorphic in *Cyclura cychlura cychlura* (Colosimo et al., 2014). PCR amplification was performed in a 13.75 µl total volume containing 10 ng of template DNA, 5 pmol of fluorescently-labeled forward primer, 5 pmol of unlabeled reverse primer, and 0.3 U Ampli*Taq*^®^ Gold (Applied Biosystems, Carlsbad, CA, USA). Thermal cycling conditions were as follows: 5 min initial denaturation at 96 °C, 35 cycles of 94 °C for 30 s, *T*_*a*_ °C (primer specific) for 30 s 72 °C for 30 s, and a final extension at 72 °C for 10 min.

**Table 3:** Twelve microsatellite loci developed for *Cyclura* spp. that were screened for polymorphism in *Cyclura cychlura inornata* using the specified annealing temperatures (*T*_*a*_).

Locus	Source species	Reference	*T*_*a*_ (°C)	Number of alleles
F436	*C. cychlura figginsi*	Malone et al., (2003)	50.0	3
F478	*C. cychlura figginsi*	Malone et al., (2003)	50.0	3
F519	*C. cychlura figginsi*	Malone et al., (2003)	55.0	2
F637	*C. cychlura figginsi*	Malone et al., (2003)	54.5	4
F2102	*C. cychlura figginsi*	Malone et al., (2003)	49.3	2
CIDK177	*C. carinata*	Welch et al., (2011)	54.8	3
60HDZ13^a^	*C. nubila*	An et al., (2004)	59.0	N/A
60HDZ151^a^	*C. nubila*	An et al., (2004)	53.3	N/A
C6	*C. pinguis*	Lau et al., (2009)	54.2	3
C124^a^	*C. pinguis*	Lau et al., (2009)	49.3	N/A
D111^b^	*C. pinguis*	Lau et al., (2009)	54.4	1
D136	*C. pinguis*	Lau et al., (2009)	51.2	3

**Notes.**

a Poor amplification

b Monomorphic

Amplified products were visualized on a 1% agarose gel using GelRed™ (Biotium, Inc., Hayward, CA, USA) and microsatellite genotyping was performed using an ABI 3100 DNA Analyzer with GeneScan™ 500 LIZ^®^ size standard. Alleles were identified using GeneMarker^®^ (SoftGenetics, State College, PA, USA).

### Data analysis

All mtDNA sequences were aligned against published sequences available from Genbank (Malone et al., 2000) using Clustal-W (Thompson, Higgins & Gibson, 1994) implemented in MEGA v. 6.0 (Tamura et al., 2013). We analyzed the full concatenated dataset, consisting of 2,328 bp of mitochondrial genes NADH-4, cytB and the control region, in a maximum-likelihood (ML) framework using RAxML v. 8.1.15 (Stamatakis, 2014). The data were partitioned by locus and analyzed under the general time-reversible (GTR) substitution model (Lanave et al., 1984) with rate heterogeneity across sites modeled by a Γ distribution and four discrete rate categories (Yang, 1994). We performed 100 ML tree searches starting with a random stepwise-addition maximum parsimony tree, followed by 1,000 bootstrap replicates (Felsenstein, 1985).

Genetic diversity measures, including allelic richness (*α*), Hardy-Weinberg Equilibrium (HWE), tests of linkage disequilibrium, F-statistics based on Weir & Cockerham (1984), expected heterozygosity (*H*_*E*_), and observed heterozygosity (*H*_*O*_), were calculated using the program GENEPOP v. 4.1 (Raymond & Rousset, 1995; Rousset, 2008). Markov Chain Monte Carlo (MCMC) parameters for GENEPOP calculations were 10,000 iterations and 1,000 batches. Allelic richness (*α*) corrected for unequal sample sizes was calculated using the rarefaction method (Hurlbert, 1971; El Mousadik & Petit, 1996; Leberge, 2002) with the program FSTAT v. 2.9.3.2 (Goudet, 1995). Correlations between population size (N) and genetic diversity measures (*α*, *H*_*E*_, *H*_*O*_, *N*_*e*_), as well as between island size and genetic diversity measures (*α*, *H*_*E*_, *H*_*O*_) (Frankham 1996) were computed using IBM SPSS Statistics (IBM, Somers, NY, USA). One-tailed tests for significance were determined at *α* = 0.05 (Frankham 1996). The relationship between geographic distance and genetic distance (*F*_*ST*_) was tested for significance with a Mantel test using the Isolation by Distance Web Service (Jensen, Bohonak & Kelley, 2005). Geographic distance was measured as the shortest distance between island pairs and log transformed according to Rousset (1997).

To estimate contemporary *N*_*e*_ in sampled cays we used *N*_*e*_*-Estimator* (Do et al., 2014). The software takes advantage of a single-time sampling event, multi-locus genotypes, and three different algorithms to estimate the parameter: the Linkage Disequilibrium, or LD method (Waples & Do, 2008); the Heterozygous Excess, or Het_ex_ method (Pudovkin, Zaykin & Hedgecock, 1996; Pudovkin, Zhdanova & Hedgecock, 2010; Zhdanova & Pudovkin, 2008); and the Coancestry method (Nomura, 2008). The LD method is based on the Burrow’s Δ estimates of LD, which can account for small sample sizes and alleles with low frequency, to produce a correlation coefficient for each locus and allele in the sample. The correlation coefficients were then used to estimates *N*_*e*_ values (Waples & Do, 2008). The Het_ex_ is based on the excess heterozygous offspring expected from the mating between parents with differences in allele frequencies (Pudovkin, Zaykin & Hedgecock, 1996). The Coancestry method is based on the estimates of a molecular coancestry parameter. This estimate of the effective number of breeders is generated from the average probability that alleles at loci in two individuals at generation *t* are inherited from the same individual in generation *t-1* (Nomura, 2008). When estimating *N*_*e*_ using the LD and Het_ex_ methods, only alleles with frequencies greater than 0.05 were considered to prevent biases associated with low allele sample sizes.

Individuals were assigned to genetic clusters using the Bayesian clustering algorithm implemented in STRUCTURE v. 2.3.4 (Pritchard, Stephens & Donnelly, 2000; Hubisz et al., 2009). Using an admixture model with no priors, a total of 10^6^ MCMC iterations were run, and the first 100,000 replicates were discarded as burn-in. Given that seven cays were sampled, we hypothesized 1 ≤ *K* ≤ 10 possible populations and performed 10 replicates of the MCMC run for each *K* value. Allowing for *K* > 7 increased our chances of discovering genetic structure within cays if it were to exist. The most likely number of clusters was estimated using the Evanno method, based on the second order of difference in likelihood function of K and implemented in the web tool STRUCTURE—HARVESTER (Evanno, Regnaut & Goudet, 2005).

## Results

We collected samples from a total of 285 individuals of *C. c. inornata* from all known inhabited cays (Table 1). On Allen and Second Cays all known individuals had been captured and marked resulting in a unique opportunity to characterize complete genetic diversity for these localities. Despite small numbers of individuals for these sites, we included these data in genetic analyses based on the expected influence of complete sampling on estimates of allelic richness and other parameters when using a rarefaction approach (Leberge, 2002).

Extractions for 15 of 285 individuals (5%) did not produce high enough quality DNA for sequencing and genotyping and were excluded from further analyses. Removal of individuals of unknown locality and poor quality DNA left 268 individuals available for analysis. Our alignment of concatenated mitochondrial sequences for NADH-4, cytochrome b and the control region for the subsample of *C. c. inornata* (*n* = 95) resulted in a single haplotype (Genbank accession KM275472, KM275473 and KM275474). Phylogenetic analysis of our data with published *Cyclura* sequences resulted in a combined *C. c. inornata* and *C. c. figginsi* subclade as in Malone et al. (2000).

Of the 12 microsatellite loci screened for polymorphisms, eight (F436, F478, F519, F637, F2102, CIDK177, C6, D136) amplified and were variable, one locus (D111) amplified but was monomorphic, and three primer pairs (60HDZ13, 60HDZ151, C124) did not produce consistent PCR products and were therefore not included in further study (Table 3). Two loci, F436 and F478, were found to exhibit evidence for significant linkage disequilibrium (*p* = 0.009) in a subset of populations. However, as the pattern was not consistent across all populations, we chose to include both loci in our final analyses.

In total, 23 distinct alleles were detected across eight polymorphic microsatellite loci and allelic richness (*α*) ranged from 1.76 to 2.09 (Table 1). Across all localities examined, no significant deviations from Hardy-Weinberg Equilibrium were detected. However, estimates of *F*_*IS*_ were negative for all islands except the small, all-female sample from Southeast Flat Rock Reef (SEFRR) Cay. Among islands, expected heterozygosity (*H*_*E*_) ranged from 0.13 to 0.46, and observed heterozygosity (*H*_*O*_) ranged from 0.09 to 0.58. The population from SEFRR Cay exhibited both the lowest *H*_*E*_ and *H*_*O*_. When this site was excluded, the ranges of *H*_*E*_ and *H*_*O*_ were 0.35–0.46 and 0.38–0.58, respectively. Effective population sizes were low relative to estimated census sizes. In the two presumed natural populations, parametric estimates ranged from 16.4 to 105, and 95% confidence intervals had variable if not infinite ranges (Table 4). Estimates of *N*_*E*_ for the translocated populations were also considered. However, sample sizes were typically low, and assumptions of these methods were not likely met given the uncertain demographic history of these populations.

**Table 4:** Effective population size (*N*_*E*_) estimates for the two natural populations (*), and the five translocated populations sampled. *N*_*E*_ was estimated using the Linkage Disequilibrium Method (LD), the Heterozygous Excess method (Het_ex_), and the Coancestry method (Co). Confidence intervals (CI, 95%) on the three estimates are also provided.

Cay	LD	LD CI	Het_*ex*_	Het_*ex*_**CI**	Co	Co CI
Leaf*	40	13.8–185	73.4	6.6–∞	27.8	0.00–139
U*	105	27.4–∞	16.4	4.6–∞	21.9	0.00–110
Alligator	48.6	1.20–∞	3.5	1.90–46.5	30	0.00–151
FRR	27.4	8.3–389	5.9	3.0–∞	∞	∞
Allen	22.6	2.3–∞	48.8	2.4–∞	10.1	0.00–50.6
Second	∞	∞	1.8	1.10–5.40	∞	∞
SEFRR	∞	∞	∞	∞	2.2	1.60–3.00

Pairwise *F*_*ST*_ values ranged from 0.01 to 0.30 with a mean of 0.17 (Table 5). The population on U Cay exhibited *F*_*ST*_ values greater than 0.15 with all other populations except Flat Rock Reef Cay (Fig. 1). The population on Leaf Cay exhibited *F*_*ST*_ values greater than 0.15 with populations on U and SEFRR Cays and *F*_*ST*_ values less than 0.15 with the other populations.

**Table 5:** Pairwise *F*_*ST*_ (Weir & Cockerham, 1984) values among sampled populations of *Cyclura cychlura inornata* spanning their current range in the Bahamas (GENEPOP v 4.1; Raymond & Rousset, 1995; Rousset, 2008).

Cay	Leaf	U	Alligator	FRR	Allen	Second
U	0.163					
Alligator	0.050	0.224				
FRR	0.121	0.016	0.177			
Allen	0.007	0.177	0.063	0.134		
Second	0.101	0.264	0.172	0.198	0.076	
SEFRR	0.156	0.298	0.228	0.276	0.183	0.426

**Figure 2:**
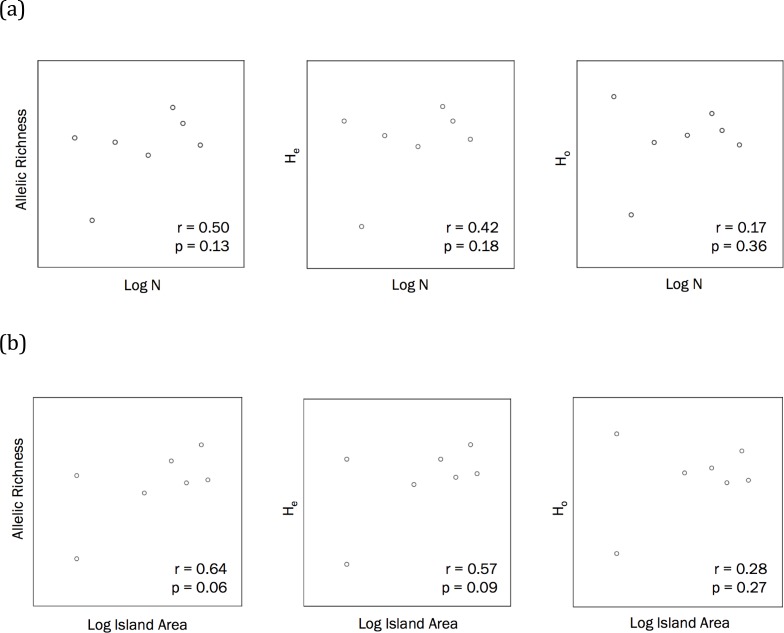
(A) Correlations to assess the relationship between Allen Cays Rock Iguana population size (*N*) and genetic diversity measures (allelic richness, expected heterozygosity (*H*_*E*_), observed heterozygosity (*H*_*O*_)) (alpha level = 0.05, *n* = 7). (B) Correlations between island area and allelic richness as well as island area and expected heterozygosity (alpha = 0.05).

**Figure 3:**
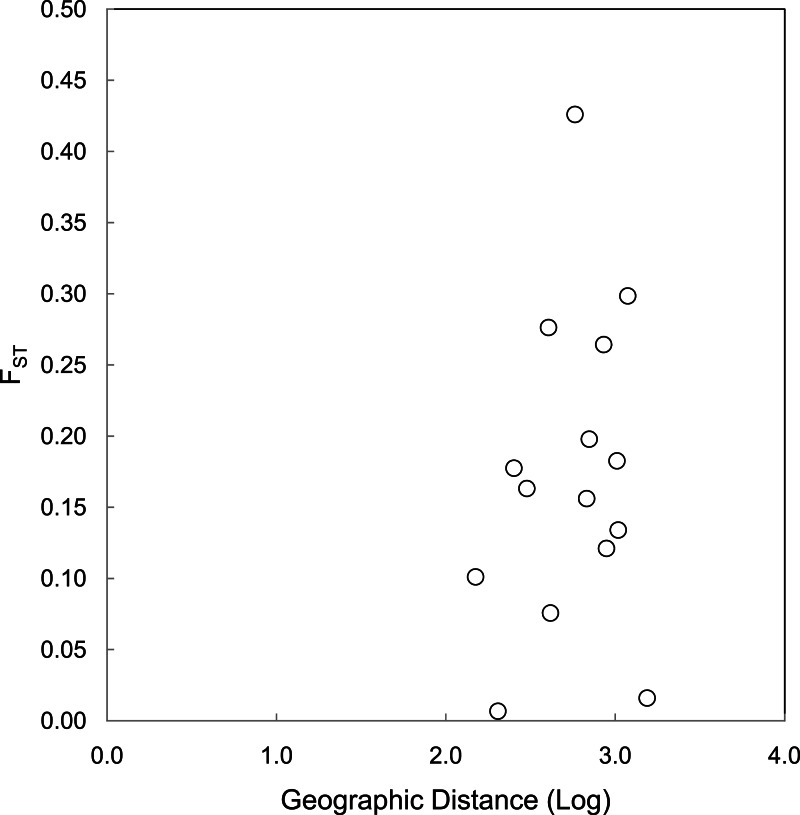
Mantel test for a correlation between geographic distance and genetic distance (FST) for Allen Cays Rock Iguana (*p* = 0.33, *r* = 0.19).

The correlations between population size and genetic diversity measures (*α*, *H*_*E*_, *H*_*O*_) were not significant (*p* = 0.13, 0.36, 0.18, respectively; Fig. 2), nor were the correlations between island area and *H*_*O*_ significant (*p* = 0.27). While not significant, the relationships between island area with *α*(*p* = 0.06) and *H*_*E*_(*p* = 0.093) were both positive. According to the Mantel test (Fig. 3), the relationship between geographic distance and genetic distance was not significant (*p* = 0.33). Prior to the test, Alligator Cay was removed from this analysis due to its history as a confirmed human-introduced population.

**Figure 4:**
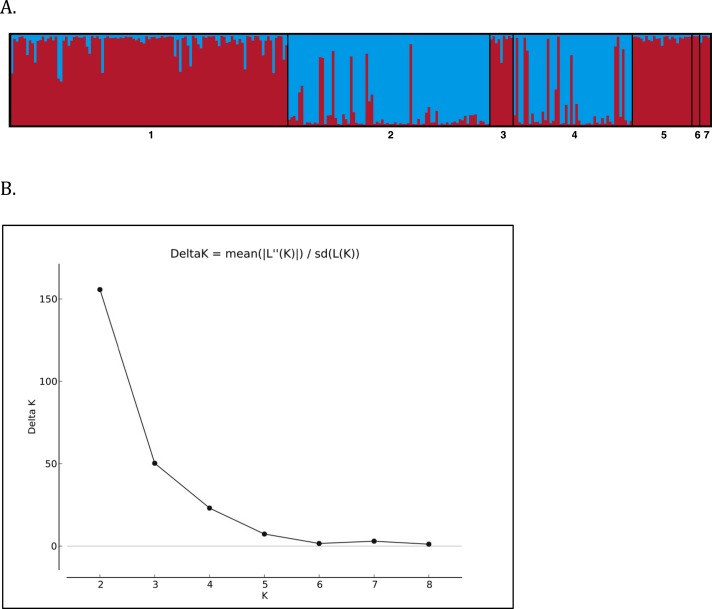
(A) STRUCTURE barplot (*k* = 2) using 8 microsatellite loci for the Allen Cays Rock Iguana *(Cyclura cychlura inornata*). 1 = Leaf, 2 = U, 3 = Alligator, 4 = Flat Rock Reef, 5 = Allen, 6 = Second, 7 = SE Flat Rock Reef. Alligator Cay (3) is an experimentally translocated population from Leaf (1). (B) Delta K values for *K* = 1--9 indicating highest likelihood at *K* = 2.

Structure Harvester indicated that the greatest change in DeltaK occurred at *K* = 2, suggesting two unique genotypic clusters (Fig. 4), with iguanas collected from the two naturally occurring populations of Leaf Cay and U Cay segregating in each cluster respectively. Individuals sampled from Leaf Cay and U Cay exhibited greater than 90% probability of assignment to their respective genetic populations; however, six individuals on U Cay exhibited a high probability (>75%) of genetic clustering with individuals sampled on Leaf Cay. Additionally, individuals from the translocated population on Alligator Cay clustered (>75% probability) with individuals on Leaf Cay, as did individuals sampled from Allen Cay, Second Cay, and Southeast Flat Rock Reef Cay (>90%). In contrast, individuals sampled on Flat Rock Reef Cay exhibited a high probability (>80%) of clustering with either U Cay or Leaf Cay.

## Discussion

Combined analysis of mitochondrial DNA and nuclear DNA microsatellite markers revealed low levels of genetic variation within *C. c. inornata*. This contrasts with several other recent analyses, including that by An et al. (2004) who screened 20 microsatellite loci in *Cyclura nubila* and found a greater range in number of alleles per locus, and Mitchell et al. (2011) who screened 23 loci in *C. pinguis* and detected three to five alleles per locus. Although these studies examined more loci, it is noteworthy that seven of the eight loci screened in our study possessed only two or three unique alleles and only one locus possessed four. This is consistent with the low levels of variation in *C. c. inornata* previously observed by Knapp & Malone (2003). Overall, allelic richness for *C. c. inornata* was 2.03, with a range of 1.31–2.09 among cays, suggesting much lower diversity than the closely related Andros Island Rock Iguana (*C. c. cychlura*) that exhibited a per cluster allelic richness ranging from 3.04 to 3.84 (Colosimo et al., 2014). Levels of allelic richness in the Allen Cays Rock Iguana were most similar to that exhibited by the conspecific Exuma Island Rock Iguanas (*C. c. figginsi*; 1.63–2.75) in the southern region of the same island chain (Malone et al., 2003).

The reduced genetic diversity of the Allen Cays Rock Iguana is notable relative to other insular reptiles studied thus far. For example, genetic analysis of the Galápagos Land Iguana (*Conolophus subcristatus*) revealed allelic richness values of 5.55 and 4.78 for two separate island populations (Fabiani et al., 2011) after a severe reduction in population size due to harvest. The endemic New Zealand Tuatara (*Sphenodon* sp.) exhibited 84 unique alleles across six microsatellite loci and allelic richness ranged from 2.2 to 5.6 in 14 island populations (MacAvoy et al., 2007). Whitakers Skink (*Cyclodina whitakeri*) from insular New Zealand also exhibited relatively high diversity with 114 unique alleles across twelve microsatellite loci (Miller et al., 2009).

Observed heterozygosity in the Allen Cays Rock Iguana (0.38–0.58), excluding the small all-female population on Southeast Flat Rock Reef Cay, are comparable to those for Anegada Iguanas (0.27–0.88, Mitchell et al., 2011) and Galápagos Land Iguanas (0.38–0.67, Gentile et al., 2009), despite relatively low allelic richness values. The low allelic richness and genetic diversity in the Allen Cays Rock Iguana is consistent with expectations given the small population sizes, small island sizes, and marked range fragmentation (Frankham, 1997; Willi, Griffin & Van Buskirk, 2013). Further, many if not all of these populations are believed to have passed through one or more bottlenecks in their recent histories.

Leaf and U Cays host the only two populations of *C. c. inornata* thought to be natural. The remaining five populations are likely the result of translocations. The two natural populations have significantly different allele frequencies (pairwise *F*_*ST*_ = 0.16). This suggests that these two populations have been isolated since rising sea-levels associated with the current interglacial partially inundated the Great Bahama Bank, a finding consistent with observations made regarding *C. c. figginsi* (Malone et al., 2003). If there is ongoing gene flow between these two Allen Cay populations it is clearly insufficient to prevent divergence between them. Colosimo et al. (2014) found that narrow water channels on Andros Island were sufficient to impede gene flow in *C. c. cychlura*. Hence, it is not surprising that we find evidence for divergence, and limited gene flow where Cays are separated by a fast flowing tidal channel that is more than 300 m wide. Further, iguanas have never been observed naturally dispersing among these islands during 35 years of fieldwork, nor are we aware of any anecdotal accounts by residents familiar with these islands (J Iverson, 2006–2016, unpublished data). However, our data suggest that unauthorized translocations of iguanas among some islands are highly likely (Smith & Iverson, 2006; J Iverson, 2006–2016, unpublished data). Populations on U Cay and Flat Rock Reef Cay have very similar allele frequencies (pairwise *F*_*ST*_ = 0.016), yet they are separated by the greatest geographic distance, excluding Alligator Cay which is known to result from translocation.

A Mantel test for geographic distance and *F*_*ST*_ excluding the Flat Rock Reef Cay population illustrated a positive trend and implied a stronger correlation, but was not significant (*p* = 0.10, *r* = 0.60). Long-term capture records identifying the presence of individuals on Flat Rock Reef Cay that were previously marked on U Cay (most) or Leaf Cay (J Iverson, 2006–2016, unpublished data) are suggestive of such unauthorized translocations.

Although the genetic diversity of *C. c. inornata* was relatively low compared to other studied *Cyclura* species, genetic structure was still evident among populations. Overall, Bayesian STRUCTURE analyses revealed that two unique genotypic clusters exist within the subspecies and mirrored patterns of genetic diversity described above (Fig. 4). The majority of individuals on Leaf and U Cay were assigned to unique clusters with high probability and likely provide the source of individuals for the surrounding cays. All the individuals on Alligator Cay were assigned to the same cluster as individuals on Leaf Cay, which is consistent with the fact that the Alligator Cay population was founded by individuals from Leaf Cay (Knapp, 2001). Moreover, the low *F*_*ST*_ value between Alligator and Leaf Cay (0.048) clearly reflects the recent (1988–1990) translocation event.

Small, discrete populations eventually experience inbreeding and loss of genetic variation (Frankham, 1995). Effective population size (*N*_*E*_), defined as the actual number of breeding individuals in a population, is predicted to influence the rate at which this loss is expected to happen (Crow & Kimura, 1970). Hence, estimating a parameter such as *N*_*E*_ is important for conservation practices. Our results indicate that effective population sizes are low for the two naturally occurring populations (Table 4). Bottlenecks and founder events could have resulted in this pattern. Because of the intensive mark-recapture work over the past 35 years, population size estimates for the natural populations are particularly robust (Iverson et al., 2006). Contrasting *N*_*E*_ estimates in *C. c. inornota* with these population size estimates allowed us to predict the effects of the species’ population ecology on its capacity to maintain genetic variation. Although *N*_*E*_ is typically much lower than actual population size, averaging three estimates of N:*N*_*E*_ for both of the natural populations suggesting that this ratio is extremely high (17.4 for Leaf Cay, and 11.6 for U Cay). Factors likely responsible for the high N:*N*_*E*_ in *C. c. inornata* include largely overlapping generations, and highly variable reproductive success. Further, the abundance of juveniles in the population likely influences N:*N*_*E*_ because the age of maturity in *C. c. inornata* is 12 years, the longest known for a lizard (Iverson, Hines & Valiulis, 2004).

Population size and habitat area have been positively linked to genetic variation and evolutionary potential (Frankham, 1996). Our results suggest a positive relationship between these parameters, although the correlations are not statistically significant. Low levels of genetic diversity, low variation in island size, and the confounding effects of historic and ongoing anthropogenic factors including translocations likely hinder our ability to detect such correlations (Van Treuren et al., 1993). In addition, the current phylogeny for the genus *Cyclura* places *C. cychlura* as the most recently derived species (Malone et al., 2000; Starostová, Rehák & Frynta, 2010). During the last glacial maximum approximately 18,000 years ago, Andros Island and the Exuma Island chain were a single contiguous land mass, and only with a subsequent rise in sea level did the islands fragment to the degree they are today (Malone et al., 2003). Combined, these findings imply that the low genetic diversity associated with a young species may have been further attenuated by the reduction in population size resulting from rising sea levels. Similar analysis of mtDNA sequences revealed shallow divergence in Whitaker’s Skink (*Cyclodina whitakeri*), which also is endemic to islands once joined as a single landmass during the last glacial maximum (Chapple, Daugherty & Ritchie, 2008).

The low genetic diversity in the Allen Cays Rock Iguana could reflect geologic history, recent anthropogenic effects, or a combination, and these factors warrant additional investigation. In particular, the most dramatic impact on populations of the Allen Cays Rock Iguana may be sourced to the extreme overharvesting of the species at the beginning of the twentieth century. Barbour & Noble (1916) declared “the species [sic] *inornata*, which once doubtless existed on several islands about Allen’s Harbor, is now beyond doubt extinct. Since these creatures are excellent for food, they are constantly hunted…often with dogs trained for the purpose.”

Rapid decreases in population size may result in rapid loss of genetic diversity, which subsequently predisposes populations to genetic drift and inbreeding (Wright, 1940). As previously discussed, the Allen Cays Rock Iguana exhibits relatively low allelic richness, but comparable levels of heterozygosity to that in other species. This finding is consistent with previous studies illustrating that measures of allelic richness are sensitive to population bottlenecks (Leberge, 2002).

### Conservation

Molecular data can augment data from ecological and behavioral research to benefit those managing wild populations. Our preliminary analyses of nuclear and mitochondrial markers in *C. c inornata* revealed low levels of genetic diversity. However, small samples sizes for some populations and historical influences make interpretation of these results difficult. Biogeography and historical and current anthropogenic factors are all suspected to have contributed to observed patterns.

For future planning, it is important to recognize that high levels of natural inbreeding in this insular species in combination with anthropogenic and environmental stressors could send the species into an extinction vortex (Caughley, 1994; Brook, Sodhi & Bradshaw, 2008). Consistent with this interpretation are the number of other natural populations of *C. cychlura* between the current ranges of *inornata* and *figginsi* in the Exumas that have gone extinct (Schwartz & Carey, 1977).

The maintenance of genetic diversity is an important component of conservation planning. Expected increases in hurricane activity and sea level rise under projected climate change scenarios pose significant threats to the persistence of *C. c. inornata*. Our evidence for genetically distinct natural populations on U Cay and Leaf Cay supports their treatment as separate management units. However, it appears that animals from Leaf Cay have already been translocated without authorization to U Cay, potentially compromising any genetic distinction of the latter population. Hence, additional translocations to historically occupied sites may still be warranted in order to maximize the preservation of remaining genetic diversity. In addition, these sites could provide beneficial assurance populations in the event of stochastic extirpations.

## Supplemental Information

10.7717/peerj.1793/supp-1 Supplemental Information 1 Cyclura cychlura inornata D-loop, cytochrome b (Cytb), and NADH dehydrogenase subunit 4 (ND4) gene region sequences

## Additional Information and Declarations

### Field Study Permissions

The following information was supplied relating to field study approvals (i.e., approving body and any reference numbers): Samples were collected and processed under permits to JI from the Bahamas National Trust, the Bahamas Environment Science and Technology (BEST) Commission, the Exuma Cays Land and Sea Park, and the Bahamas Department of Agriculture.

### DNA Deposition

The following information was supplied regarding the deposition of DNA sequences: Genbank accession numbers KM275472, KM275473 and KM275474.
